# Supplementary material for: Generation of PVP Membranes Using Extracts/Phenolic Fraction of Dysphania ambrosioides, Opuntia ficus-indica, and Tradescantia pallida
Source: Polymers (Basel). 2023 Dec 15;15(24):4720. doi: 10.3390/polym15244720 (PMC10747495; doi:10.3390/polym15244720)
Supplement: Supplementary file 1 [file polymers-15-04720-s001.zip › polymers-2746379-supplementary.pdf]

Supplementary data

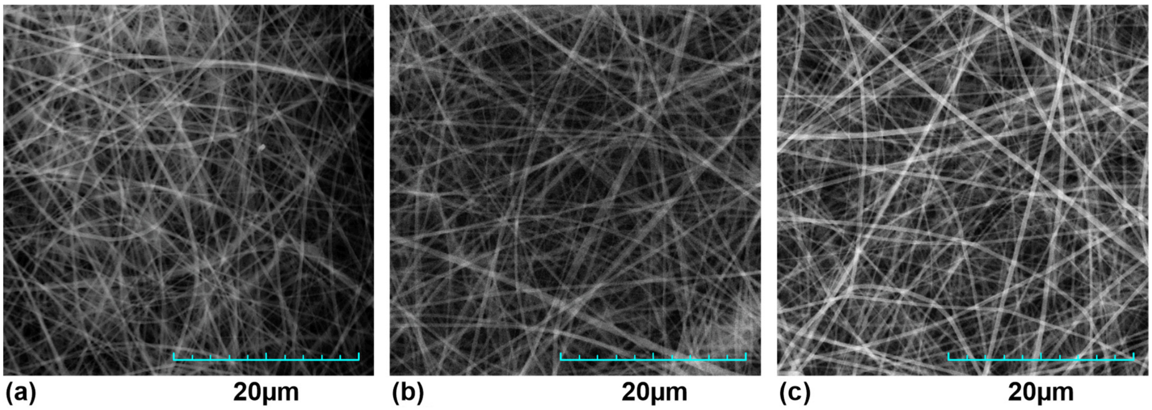

Figure S1. SEM micrograph of membranes. (**a**) FR-DA+PVP, (**b**) FR-OFI+PVP, and (**c**) FR-TP+PVP.

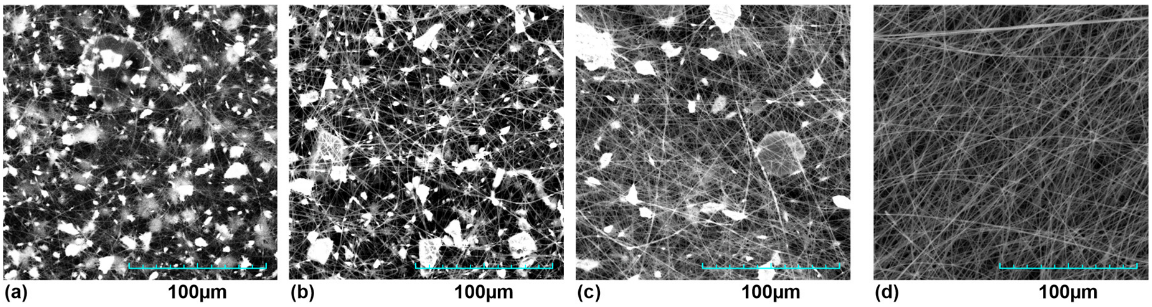

Figure S2. SEM micrograph of membranes. (**a**) DA+PVP, (**b**) OFI+PVP, (**c**) TP+PVP, and (**d**) PVP Control

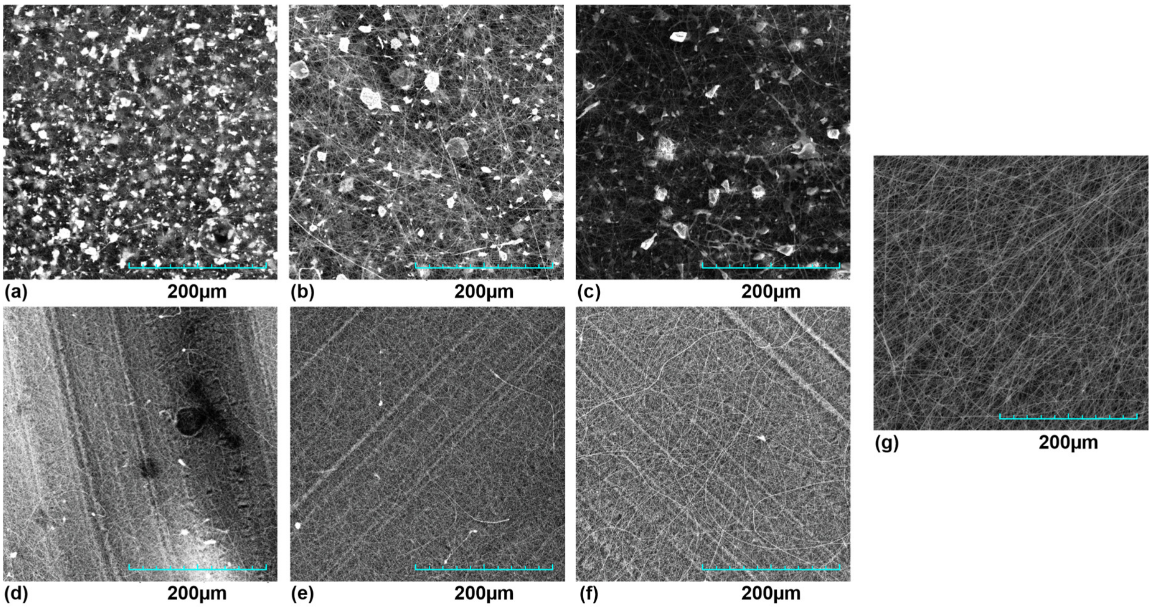

Figure S3. SEM micrograph of membranes. (**a**) DA+PVP, (**b**) OFI+PVP, (**c**) TP+PVP, (**d**) FR-DA+PVP, (**e**) FR-OFI+PVP, (**f**) FR-TP+PVP, and (**g**) PVP Control

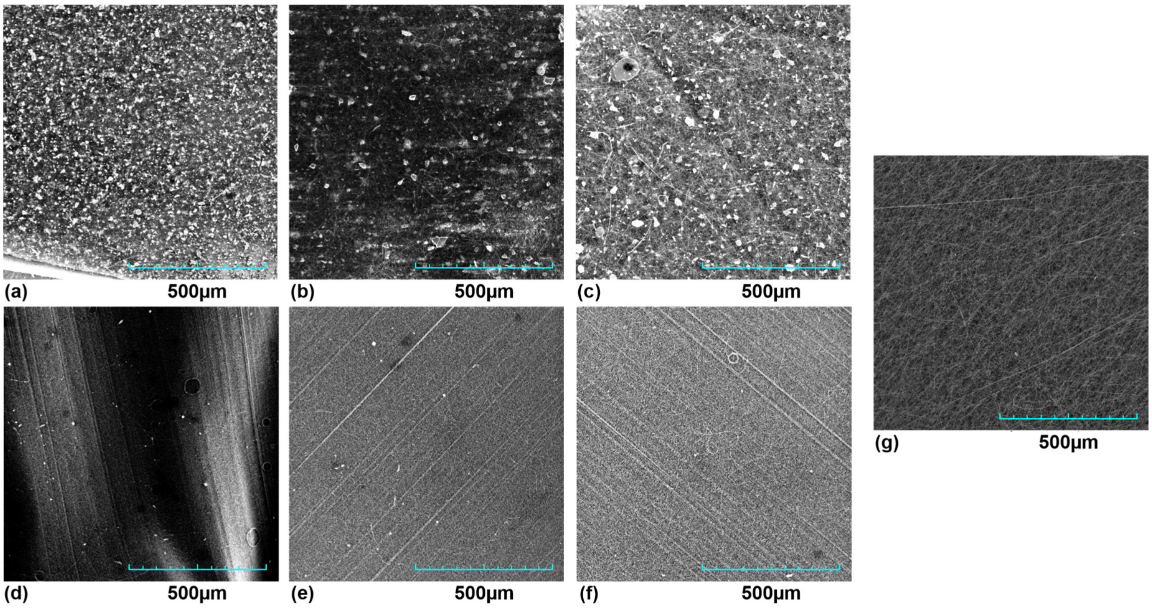

Figure S4. SEM micrograph of membranes. (**a**) DA+PVP, (**b**) OFI+PVP, (**c**) TP+PVP, (**d**) FR-DA+PVP, (**e**) FR-OFI+PVP, (**f**) FR-TP+PVP, and (**g**) PVP Control
